# Prognostic Factors and Survival Outcomes in Pediatric Acute Lymphoblastic Leukemia: A Real-World Retrospective Cohort Study from a Tertiary Care Center in North India

**DOI:** 10.7759/cureus.112537

**Published:** 2026-07-12

**Authors:** Faisal R Guru, Suhail Ahmad Wani, Shumail Bashir, Nisar Ahmad Syed, Mohmad Hussain Mir, Ulfat Ara, Tazeen Jeelani, Zafirah Zahir, Aiffa Aiman

**Affiliations:** 1 Department of Medical Oncology, Sher-i-Kashmir Institute of Medical Sciences, Srinagar, IND; 2 Department of Chest Medicine, Government Medical College (GMC) Baramulla, Srinagar, IND; 3 Department of Pathology, Sher-i-Kashmir Institute of Medical Sciences, Srinagar, IND

**Keywords:** acute lymphoblastic leukemia (all), bfm-95 protocol, cytogenetics, kashmir, low- and middle-income countries, minimal residual disease, pediatric all, prognostic factors, risk stratification, survival outcomes

## Abstract

Background

Acute lymphoblastic leukemia (ALL) is the most common childhood malignancy, with outcomes varying significantly across different populations. While survival rates have significantly improved in high-income countries, data from low- and middle-income countries (LMICs) remain heterogeneous. The Kashmir region in India represents a unique population with distinct genetic and demographic characteristics, including high rates of consanguinity and potential pharmacogenomic variability. This study aimed to evaluate the prognostic significance of clinical and biological factors and their impact on survival outcomes in pediatric ALL patients treated in a real-world tertiary care setting.

Methods

This retrospective cohort study included pediatric patients (aged 1-18 years) diagnosed with ALL between June 2016 and November 2023 at the State Cancer Institute, Sher-i-Kashmir Institute of Medical Sciences, Srinagar, Jammu & Kashmir, India. All patients were treated according to the Berlin-Frankfurt-Münster (BFM)-95 pediatric ALL protocol with risk stratification based on clinical parameters, cytogenetics, and early treatment response. Key prognostic variables included age, gender, leukocyte count at presentation, immunophenotype, cytogenetics, Day 8 prednisolone response, and Day 33 bone marrow and minimal residual disease (MRD) status post induction. Survival outcomes were assessed using Kaplan-Meier analysis, and prognostic factors were evaluated using univariate and multivariate Cox regression models.

Results

A total of 232 patients were analyzed after exclusions. The median age was 8.5 years, with a male predominance (58.2%, n=135). B-cell ALL constituted (84.9%, n=197) of cases. A good prednisolone response on Day 8 was observed in 96.6% (n=224) of patients. On day 33, 89.7% (n=208) achieved bone marrow remission and 82.8% (n=192) attained MRD negativity. After a median follow-up of 49 months, 43 patients (18.5%) relapsed, and 56 patients (24.1%) died. The estimated five-year overall survival (OS) and event-free survival (EFS) were 74.5% and 66.9%, respectively. On univariate analysis, favorable prognostic factors included younger age, B-cell phenotype, absence of high-risk cytogenetics, MRD negativity, and marrow remission at Day 33. On multivariate analysis, *MLL* (mixed lineage leukemia) gene rearrangement, MRD positivity, lack of marrow remission at Day 33, and CNS involvement were independent predictors of inferior survival.

Conclusion

This study demonstrates that outcomes of pediatric ALL in Kashmir are comparable to those in other resource-limited settings and confirms the pivotal role of early treatment response, particularly MRD status, in prognostication. Despite unique genetic and demographic factors, BFM-based risk-adapted therapy remains effective in this population. These findings underscore the need for enhanced access to molecular diagnostics and MRD assessment to further optimize outcomes and enable personalized therapy in this region.

## Introduction

Acute lymphoblastic leukemia (ALL) is the most common childhood malignancy, accounting for approximately 25-30% of all pediatric cancers. Over the past few decades, significant advances in risk stratification, supportive care, and treatment protocols have markedly improved survival outcomes, with cure rates exceeding 90% in high-income countries. However, outcomes in low- and middle-income countries (LMICs) remain heterogeneous due to differences in disease biology, access to diagnostics, treatment adherence, and supportive care [1,2].

Prognosis in pediatric ALL is influenced by several well-established clinical and biological factors, including age at diagnosis, initial leukocyte count, immunophenotype, cytogenetic and molecular abnormalities, extramedullary disease, central nervous system (CNS) involvement and response to therapy, particularly minimal residual disease (MRD) [3,4]. Risk-adapted treatment strategies based on these prognostic indicators form the cornerstone of contemporary pediatric ALL management and are recommended by international cooperative leukemia study groups such as the Children's Oncology Group (COG) and the Berlin-Frankfurt-Münster (BFM) Study Group [5,6].

Despite these advances, the distribution and prognostic significance of these factors may vary across different populations due to genetic, environmental, and healthcare-related determinants. The Kashmir region represents a unique population with distinctive genetic characteristics, including a relatively high prevalence of consanguinity and intra-community marriages. Such demographic patterns may influence genetic diversity, predisposition to certain malignancies, and the prevalence of specific cytogenetic or molecular alterations in leukemia [7]. Additionally, variations in pharmacogenomic profiles and gene polymorphisms may potentially affect treatment response, toxicity patterns, and overall outcomes in pediatric ALL [8].

Furthermore, studies from India and other developing regions have reported differences in clinical presentation, treatment outcomes, and survival patterns in pediatric ALL compared with Western populations, highlighting the importance of region-specific data [9,10]. However, data describing the clinical profile, biological characteristics, and survival outcomes of pediatric ALL from the Kashmir region remain limited.

Understanding how established prognostic indicators perform within this distinct population context is important for optimizing risk stratification and tailoring management strategies. Therefore, this study aimed to evaluate the impact of established prognostic factors on the survival outcomes among pediatric ALL patients and to evaluate the clinical and biological prognostic factors in this unique regional population. This study seeks to generate relevant clinical evidence that may contribute to improved risk stratification and a better understanding of pediatric ALL in this region and beyond.

## Materials and methods

Study design and setting

This retrospective observational study was conducted at the Department of Medical Oncology, State Cancer Institute, Sher-i-Kashmir Institute of Medical Sciences, a tertiary care referral center in Srinagar, serving the population of Jammu and Kashmir and surrounding regions in India. The study was approved by the Institutional Ethics Committee, Sher-i-Kashmir Institute of Medical Sciences (IEC/SKIMS protocol: #RP156/2022).

Study population

All pediatric patients diagnosed with ALL between June 2016 and November 2023 were screened and followed up to February 29, 2024. Eligibility criteria were defined prior to data collection to ensure uniform selection of pediatric patients with ALL (Table 1). A total of 249 patients aged 1-18 years fulfilled the eligibility criteria, and 232 were included in the final analysis, as 17 patients were excluded due to transfer to other institutions.

**Table 1:** Inclusion and exclusion criteria

Inclusion Criteria	Exclusion Criteria
Newly diagnosed acute lymphoblastic leukemia (ALL)	Infantile leukemia (<1 year of age)
Age group 1–18 years	Relapsed ALL at presentation
Diagnosis confirmed by bone marrow morphology and flow cytometry	Mixed phenotype acute leukemia (MPAL)
Both B-cell ALL and T-cell ALL were included	-
Both Philadelphia chromosome-positive and Philadelphia chromosome-negative ALL were eligible	-
Patients treated at the study institution during the study period	-

Baseline evaluation

At diagnosis, all patients underwent comprehensive baseline evaluation including complete blood counts and peripheral smear, bone marrow aspiration and morphology, immunophenotyping by flow cytometry, cytogenetic and molecular analysis where available, serum biochemical profile, and radiological evaluation when clinically indicated.

Definition of prognostic variables

Key prognostic variables were defined according to established pediatric ALL criteria used in the BFM-95 protocol, which include variables as in Table 2.

**Table 2:** Prognostic variables with standard definitions MRD: minimal residual disease; ALL: acute lymphoblastic leukemia

Prognostic Variable	Definition/Categories
Age at diagnosis	Favorable: 1–6 years; Unfavorable: <1 year or ≥6 years
Initial leukocyte count	Favorable: <20,000/mm³; High-risk: ≥20,000/mm³
Immunophenotype	B-cell precursor ALL; T-cell ALL
Early treatment response (Day 8 peripheral blast count)	Peripheral blood blast count assessed after 7 days of steroid therapy
Day-8 steroid response	Good response: <1,000 blasts/µL; Poor response: ≥1,000 blasts/µL
Bone marrow response (Day 33)	M1 marrow: <5% blasts; M2 marrow: 5–25% blasts; M3 marrow: >25% blasts
Minimal residual disease (MRD)	Assessed at Day-33 using flow cytometry, where available
MRD status	MRD negative: <0.01% leukemic cells; MRD positive: ≥0.01% leukemic cells

Risk stratification

Risk stratification was performed according to the BFM-95 pediatric ALL protocol, incorporating clinical characteristics and early treatment response (Table 3). Risk group assignment guided treatment intensity throughout therapy.

**Table 3:** BFM-95 risk stratification Risk categories were defined as per Berlin-Frankfurt-Münster (BFM-95) Study Group criteria for risk-adapted therapy strategies for childhood ALL [6]. ALL: acute lymphoblastic leukemia; WBC: white blood cell count; ABC: absolute blast count

Risk Category	Criteria
Standard Risk	Age 1–6 years, initial WBC <20,000/mm³, B-cell phenotype, good steroid response (Day 8 ABC <1000), favorable marrow response
Intermediate Risk	Patients not fulfilling criteria for Standard Risk or High Risk
High Risk	Poor steroid response (Day-8 ABC ≥1000), high leukocyte count, T-cell ALL with high tumor burden, unfavorable marrow response or persistent MRD

Treatment protocol

All patients were treated according to the BFM-95 pediatric ALL chemotherapy protocol, which consists of the following phases: induction therapy, consolidation, delayed intensification, and maintenance therapy. Treatment intensity was adapted according to the assigned risk category. Patients with Philadelphia chromosome-positive ALL received tyrosine kinase inhibitor therapy when available in addition to chemotherapy.

Response assessment

Bone marrow examination and MRD assessment were performed on Day 33 to evaluate induction response. Treatment response was assessed using standard hematologic criteria. Complete remission (CR) was defined as bone marrow blasts <5%, recovery of peripheral blood counts, MRD negative, and absence of extramedullary disease.

Supportive care

Supportive care measures included antimicrobial prophylaxis, transfusion support, management of febrile neutropenia, and treatment of chemotherapy-related toxicities according to institutional protocols. Growth factor support and nutritional support were provided where clinically appropriate.

Data collection

Clinical and laboratory data were retrieved retrospectively from hospital medical records and electronic databases. Variables collected included: demographic characteristics, baseline hematologic parameters, immunophenotype, cytogenetic and molecular findings, Day 8 prednisolone response, Day 33 bone marrow response, MRD status, treatment details, and follow-up and survival outcomes. All data were anonymized prior to analysis.

Survival outcomes

The primary endpoints were overall survival (OS) and event-free survival (EFS). OS was defined as the time from diagnosis to death from any cause or last follow-up. EFS was defined as the time from diagnosis to relapse, death, treatment failure, or last follow-up without an event. 

The secondary endpoints were to evaluate the impact of various prognostic factors on overall survival. Lost to follow-up was defined as failure to maintain scheduled follow-up and adherence to treatment protocol, with no valid clinical outcome despite repeated contact attempts.

Statistical analysis

Statistical analyses were performed using IBM SPSS Statistics for Windows, version 23 (Released 2015; IBM Corp., Armonk, New York, United States). Continuous variables were summarized using medians and ranges, while categorical variables were presented as frequencies and percentages. Survival probabilities were estimated using the Kaplan-Meier method, and comparisons between groups were performed using the log-rank test. Univariate analyses were conducted to identify prognostic factors associated with survival outcomes. Variables of clinical relevance were further explored in multivariate Cox regression models where appropriate. A p-value <0.05 was considered statistically significant. Patients with missing values for variables entered into a given mode were excluded from the specific analysis using complete-case (listwise) deletion. No imputation of missing data was performed. Patients who were lost to follow-up were censored at the date of their last documented clinical contact for OS and EFS analysis.

## Results

Patient characteristics

Of the 249 patients who met the inclusion criteria, 17 were excluded due to transfer to other institutions for further management, leaving 232 patients for final analysis. The median age was 8.5 years (range, 1-18 years), and 135 patients (58.2%) were male. B-ALL accounted for 197 patients (84.9%), while the remaining 35 patients (15.1%) had T-ALL. WBC (white blood cell) count at presentation was >20,000 in 70 patients (30.2%). Conventional karyotyping was done in 178 patients (76.7%), and among them, seven patients (3.0%) and six patients (2.6%) were hyper-diploid and hypo-diploid, respectively. Cytogenetics was done in 206 (88.6%), with 18 patients (7.8%) having *BCR-ABL* (Breakpoint Cluster Region-Abelson) and nine patients (3.9%) having *MLL* gene rearrangement. In the entire patient cohort, 113 (48.7%) were classified as standard risk, 57 (24.6%) as intermediate risk, and 62 (26.7%) as high-risk ALL based on the available parameters. CNS involvement at diagnosis was noted in 10 (4.3%) patients of the cohort. Detailed patient demographic and clinical characteristics are shown in Table 4.

**Table 4:** Baseline clinical and disease characteristics of the study cohort (N = 232) Values given as frequency (percentage) except for age at diagnosis, which is given as median (range) ALL: acute lymphoblastic leukemia; WBC: white blood cell; CNS: central nervous system

Characteristics		Frequency (Percentage)
Age at diagnosis (years)	Median (range)	8.5 (1–18)
Sex	Male	135 (58.2)
	Female	97 (41.8)
Immunophenotype	B-cell ALL	197 (84.9)
	T-cell ALL	35 (15.1)
Presenting WBC count (/mm³)	<20,000	162 (69.8)
	20,000–50,000	46 (19.8)
	>50,000	24 (10.4)
Karyotype	Normal	165 (71.1)
	Hyper-diploidy	7 (3.0)
	Hypodiploidy	6 (2.6)
	Not available	54 (23.3)
Cytogenetics	TEL-AML	36 (15.5)
	BCR-ABL	18 (7.8)
	MLL rearrangement	9 (3.9)
	Not available	26 (11.2)
CNS disease at presentation		10 (4.3)
Risk stratification	Standard risk	113 (48.7)
	Intermediate risk	57 (24.6)
	High risk	62 (26.7)

Outcomes

In the entire cohort of 232 patients who were started on the risk-allocated BFM-95 treatment protocol, 224 (96.6%) had a good prednisolone response at Day 8. On bone marrow evaluation for response assessment post induction, 208 patients (89.7%) had bone marrow in remission, while 192 (82.8%) achieved MRD negativity. After a median follow-up of 49 months (IQR, 41-61), 43 patients (18.5%) relapsed, 11 (3.8%) underwent allo-SCT (eight were MRD-positive post induction, nine had *MLL* gene rearrangement), and 56 (24.1%) died. Seven patients (3.0%) were lost to follow-up (Table 5).

**Table 5:** Treatment response and clinical outcomes of the study cohort (N = 232) CNS: central nervous system; MRD: minimal residual disease

Characteristics		Frequency (Percentage)
Treatment initiated		232 (100.0)
Day 8 peripheral blood response	Good prednisolone response	224 (96.6)
	Poor prednisolone response	8 (3.4)
Day 33 bone marrow assessment	Remission achieved	208 (89.7)
	Not in remission	24 (10.3)
Post-induction MRD status	MRD negative	192 (82.8)
	MRD positive	40 (17.2)
Pattern of relapse (Total: n=43, 18.5%)	Bone marrow relapse	27 (11.6)
	CNS relapse	6 (2.6)
	Combined bone marrow and CNS relapse	10 (4.3)
On treatment at last follow-up		50 (21.6)
Present status	Alive in remission	181 (78.0)
	Alive with relapsed disease	4 (1.7)
	On treatment	50 (21.6)
	Lost to follow-up	7 (3.0)
	Expired	56 (24.1)

**Figure 1:**
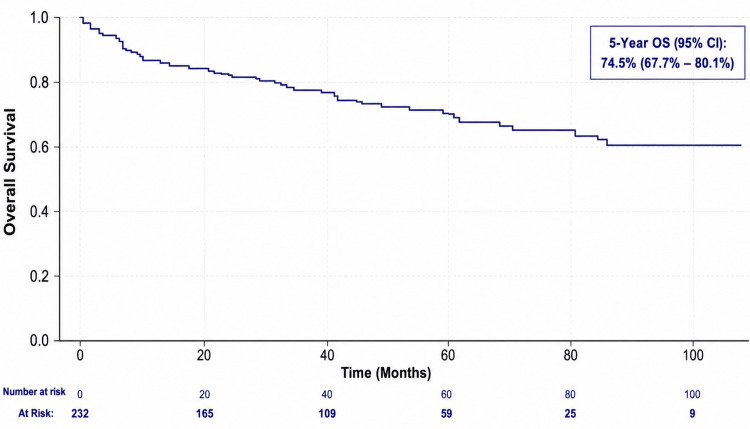
Kaplan-Meier plot of OS (months) for whole cohort. OS: overall survival; CI: confidence interval

**Figure 2:**
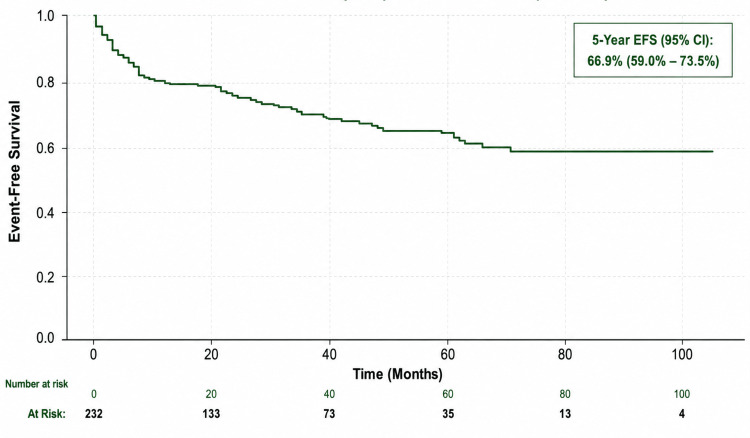
Kaplan-Meier plot of EFS (months) for whole cohort. EFS: event-free survival; CI: confidence interval

Estimated one-year, two-year, and five-year OS rates were 87.1%, 84.1%, and 74.5%, respectively (95% CI, 67.7-80.1%). EFS was 83.7%, 78.1%, and 66.9% (95% CI, 59.0-73.5%) at one, two, and five years, respectively (Figures 1, 2).

**Figure 3:**
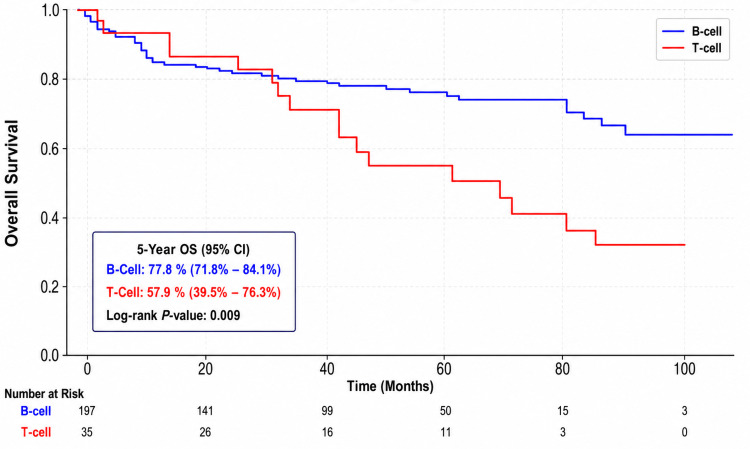
Kaplan-Meier plot of OS (months) in relation to immune-phenotype. OS: overall survival; CI: confidence interval

**Figure 4:**
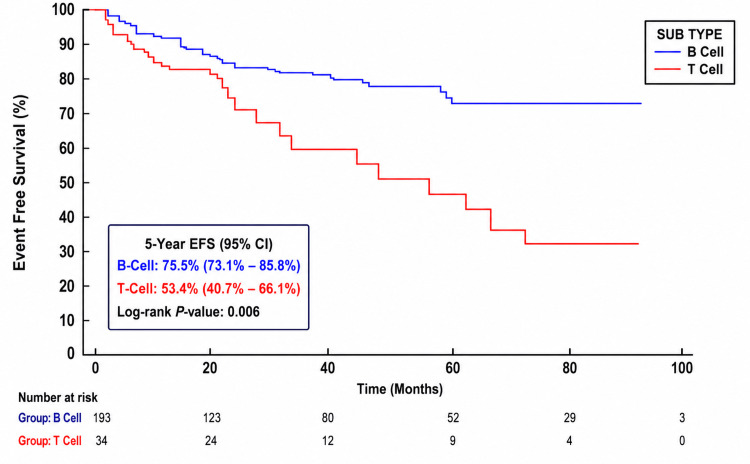
Kaplan-Meier plot of EFS (months) in relation to immune-phenotype. EFS: event-free survival; CI: confidence interval

Among 197 patients with B-ALL and 35 patients with T-ALL, the estimated five-year OS was 77.8% and 57.9%, respectively, while five-year EFS was 75.5% and 53.4%, respectively, which although statistically significant on univariate analysis, didn’t attain statistical significance on multivariate analysis (Figures 3, 4).

**Figure 5:**
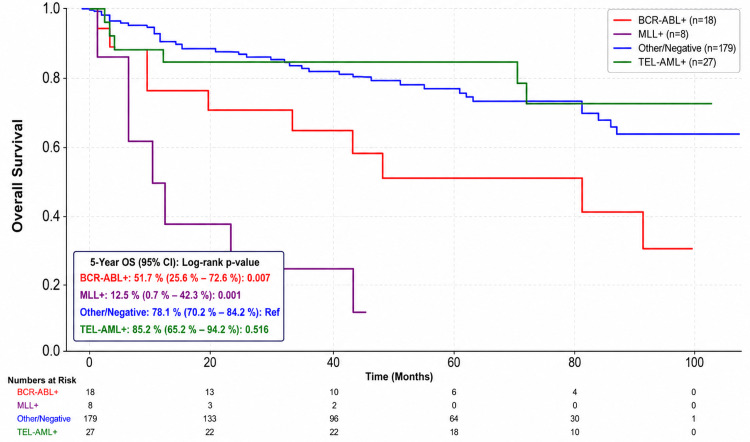
Kaplan-Meier plot of OS in relation to cytogenetic abnormalities. OS: overall survival; CI: confidence interval

**Figure 6:**
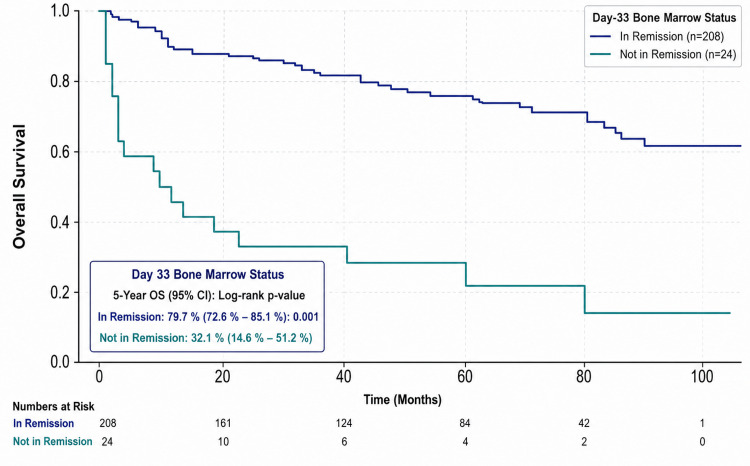
Kaplan-Meier plot of OS in relation to Day 33 bone marrow. OS: overall survival; CI: confidence interval

**Figure 7:**
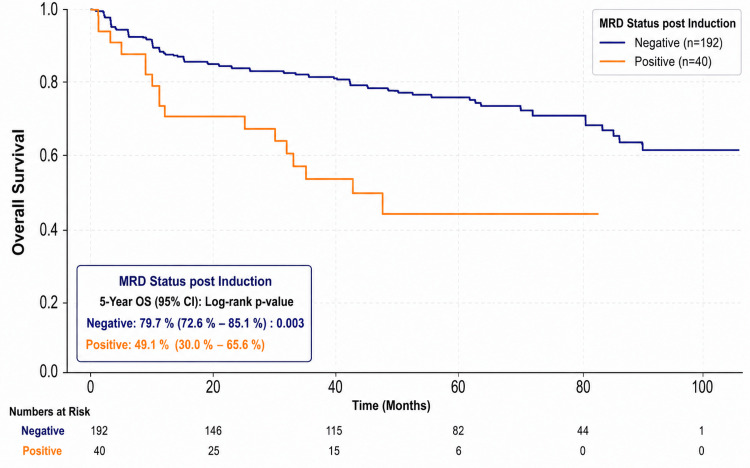
Kaplan-Meier plot of OS in relation to MRD post induction. OS: overall survival; MRD: minimal residual disease; CI: confidence interval

On univariate analysis, B cell ALL, absence of *BCR-ABL* and *MLL* gene rearrangement, Day 33 marrow in remission, MRD negativity post induction, and absence of CNS disease at presentation were all associated with significantly favorable survival (Table 6; Figures 3-7).

**Table 6:** Univariate Cox regression analysis of prognostic factors associated with survival outcomes. ALL: acute lymphoblastic leukemia; MRD: minimal residual disease; CNS: central nervous system; CI: confidence interval

Variable	Hazards Ratio (HR) [95% CI]	p-value
Age (<6 years)	1.06 [1.00, 1.12]	0.061
Immuno-Phenotype (T-cell ALL)	3.10 [1.48, 6.50]	0.003
*MLL* Gene Rearrangement (Absent)	0.20 [0.07, 0.56]	0.002
*BCR-ABL* Translocation (Absent)	0.36 [0.17, 0.76]	0.007
Day 33 Marrow (Not in Remission)	10.87 [4.07, 28.99]	< 0.001
MRD status (Positive)	2.35 [1.15, 4.77]	0.018
CNS disease (Absent)	0.23 [0.06, 0.85]	0.027

However, on multivariate analysis, only *MLL* gene rearrangement, Day 33 marrow in remission, MRD negativity post induction, and absence of CNS disease at presentation showed statistically significant survival outcome (Table 7; Figures 5-7). Due to the relatively small number of patients in the Day 33 marrow not in remission, the effect estimate should be interpreted with appropriate caution. Although outcomes appeared numerically superior in B-cell ALL immune-phenotype and younger age group, these differences did not reach statistical significance on multivariate analysis (Table 7).

**Table 7:** Multivariate Cox regression analysis of prognostic factors associated with survival outcomes MRD: minimal residual disease; CNS: central nervous system; CI: confidence interval

Variable	Hazards Ratio (HR) [95% CI]	p-value
Age (>6 years)	1.07 [1.00, 1.15]	0.067
Day 33 Marrow (Not in Remission)	12.48 [4.01, 38.91]	< 0.001
MRD status (Positive)	3.02 [1.27, 7.19]	0.012
CNS disease (Absent)	0.16 [0.03, 0.78]	0.023
*MLL *gene Rearrangement (Absent)	0.15 [0.04, 0.61]	0.008

## Discussion

In this retrospective cohort study of 232 pediatric patients with ALL treated at a tertiary care center in India, we evaluated the impact of established clinical and biological prognostic factors on survival outcomes in a real-world setting. Our findings demonstrate that outcomes in our cohort are broadly comparable to those reported from other LMICs, with a five-year OS of 74.5% and EFS of 66.9%. Importantly, early treatment response parameters, particularly Day 33 bone marrow in remission and MRD negativity, emerged as the most significant predictors of survival, while traditional baseline factors such as age, gender, immune-phenotype and leukocyte count did not show statistically significant associations.

Over the past several decades, advances in risk-adapted therapy have transformed pediatric ALL from a uniformly fatal disease to one with survival rates exceeding 85-90% in high-income settings [1-3,11]. These improvements have been largely driven by cooperative group protocols such as those developed by the COG and the BFM Group, which integrate clinical, cytogenetic, and response-based parameters into treatment stratification [5,6,12,13]. However, outcomes in LMICs remain variable due to disparities in healthcare infrastructure, treatment adherence, and supportive care [14,15]. In this context, our study provides valuable insight into the applicability of BFM-based protocols in a resource-constrained yet genetically distinct population.

The demographic and disease characteristics of our cohort are consistent with previously reported Indian and regional data. The predominance of B-cell ALL (84.9%) and male preponderance (58.2%) align with global epidemiological patterns [7,8,10]. Similarly, the proportion of patients presenting with high leukocyte counts and CNS involvement was comparable to prior studies from developing countries [9,10,15]. However, conventional karyotyping was performed in 76.7% of patients, with cytogenetic results available in 88.7%. This incomplete cytogenetic assessment underscores a common challenge in LMICs, where infrastructural and economic barriers limit access to comprehensive genomic analysis and risk stratification in hematological malignancies, reflecting gaps in optimal therapeutic allocation and thus contributing to outcome disparities compared to high-resource settings.

Early treatment response has consistently been shown to be the most powerful predictor of outcome in pediatric ALL [16-18]. In our cohort, a high proportion of patients (96.6%) demonstrated a good prednisolone response on Day 8, which is comparable to outcomes reported in BFM-based studies [12,13]. Furthermore, achievement of bone marrow remission at Day 33 and MRD negativity were strongly associated with improved overall survival, reinforcing the central role of response-adapted therapy. These findings are in concordance with large international studies demonstrating that MRD is the single most important prognostic factor in pediatric ALL [4,16-18].

In contrast, traditional baseline prognostic factors such as gender and initial leukocyte count did not demonstrate statistically significant associations with survival in our study. While there was a trend toward better outcomes in younger children and B-cell ALL immune-phenotype, these differences did not reach statistical significance on multivariate analysis. This may be attributable to the relatively small sample size or the modifying effect of risk-adapted therapy, which has been shown to mitigate the impact of baseline risk factors in contemporary treatment protocols [19-21].

The presence of adverse cytogenetic abnormalities, particularly *MLL* gene rearrangements, was associated with significantly inferior survival in our cohort, which is consistent with prior literature identifying *MLL*-rearranged ALL as a high-risk subtype with poor outcomes despite intensive therapy [22]. Similarly, although patients with T-cell ALL demonstrated numerically inferior survival compared to B-cell ALL, this difference was not statistically significant, reflecting improvements in T-ALL outcomes with modern therapy [23,24].

An important aspect of our study is the evaluation of outcomes within the unique genetic and demographic context of the Kashmir region. The relatively high prevalence of consanguinity and intra-community marriages in this population may influence the distribution of genetic variants and disease biology [7,25]. Additionally, pharmacogenomic variability may impact drug metabolism, toxicity, and treatment response [8,26]. While our study was not designed to directly assess genetic polymorphisms or treatment-related toxicity, which warrants a further prospective study, the observed outcomes suggest that standard BFM-based protocols remain effective in this population.

The relapse rate in our cohort (18.5%) and mortality rate (24.1%) are comparable to reports from other tertiary care centers in LMICs [11,15]. Notably, a subset of patients underwent allogeneic stem cell transplantation, primarily for persistent MRD positivity or high-risk disease features, reflecting adherence to risk-adapted treatment strategies. Despite these advances, treatment-related mortality and loss to follow-up remain important challenges in our setting and highlight the need for strengthening supportive care infrastructure and patient follow-up systems.

Our study has several important implications for clinical practice in the Kashmir region. First, it demonstrates that standardized treatment protocols such as BFM-95 can achieve favorable outcomes when implemented effectively in a tertiary care setting. Second, it underscores the importance of proper risk evaluation, including comprehensive genetic testing and early response assessment, particularly MRD evaluation, in guiding treatment decisions. Third, it highlights the need for region-specific data to inform risk stratification and optimize resource allocation.

However, several limitations should be acknowledged. The retrospective design of the study introduces potential biases related to data completeness and follow-up. Cytogenetic and molecular data were not available for all patients, limiting the ability to fully evaluate their prognostic impact. Chemotherapy-related toxicity, cause of mortality, and pharmacogenomic profiles were not systematically evaluated, limiting the assessment of their influence on the treatment outcomes. Additionally, MRD assessment was not uniformly available across the entire cohort. Despite these limitations, the relatively large sample size and long follow-up period strengthen the validity of our findings.

## Conclusions

This study provides comprehensive real-world data on prognostic factors and survival outcomes in pediatric ALL from a tertiary care center in northern India. Our findings confirm the critical role of early treatment response, particularly MRD negativity, in predicting survival outcomes, while also highlighting the applicability of BFM-based protocols in this unique population. These results contribute to the growing body of evidence supporting the need for region-specific data in pediatric oncology and may inform future efforts to optimize treatment strategies in similar settings.
